# Development and clinical evaluation of a quantitative fluorescent immunoassay for detecting canine CRP

**DOI:** 10.1080/23144599.2023.2247250

**Published:** 2023-08-28

**Authors:** Jawun Choi, Min-jae Yoo, Ye-Ji Jang, Byeonghak Na, Seul-ki Seo, Joungdae Moon, Jihoo Lee, Jae-won Seol

**Affiliations:** a College of Veterinary Medicine, Jeonbuk National University, Iksan, Republic of Korea; b Product Business Office, GenBody Biotech Institute, Cheonan-si, Chungcheongnam-do, Republic of Korea; c Raw Material Business Office, GenBody Biotech Institute, Cheonan-si, Chungcheongnam-do, Republic of Korea

**Keywords:** Canine, C-reactive protein, fluorescent immunoassay, correlation, rapid diagnostic test

## Abstract

Canine C-reactive protein (cCRP) is one of the major positive acute phase proteins in dogs and is commonly measured to detect and monitor systemic inflammation as well as the efficacy of treatment. Traditional methods for testing cCPR, including enzyme-linked immunosorbent assay (ELISA), have some drawbacks, such as a long time for diagnosis and the requirement of well-equipped laboratories. Therefore, there is a need for a rapid and precise diagnostic test for cCRP at point-of-care. This study assessed the accuracy, precision, and validated clinical effectiveness of a diagnostic test based on fluorescent lateral flow immunoassay to detect cCRP. For the standard cCRP concentration ranging from 0 to 200 μg/mL, the cCRP diagnostic test showed strong linearity with R^2^ of 0.9977 (*p* < 0.001), and both inter- and intra-assay CVs were <14%. The limit of detection and limit of quantitation were found to be 4.0 μg/mL and 5.0 μg/mL, respectively. The cCRP serum concentration was evaluated in 21 client-owned dogs and the results were compared to a previously validated ELISA. The Pearson Correlation Coefficient between the diagnostic test kit and ELISA was 0.942 [95% confidence interval: 0.859 to 0.976, *p* < 0.001], and the Bland–Altman plot indicated a bias of 26.82% [95% limits of agreement: −56.03 to 109.67], indicating a significant correlation and the agreement between the data from the cCRP diagnostic test and ELISA. In conclusion, the fluorescent immunoassay based diagnostic test is a suitable option for rapidly and precisely detecting cCRP in dogs, providing a convenient alternative to traditional methods for diagnosing acute inflammation.

## 1. Introduction

C-reactive protein (CRP) is a major acute phase protein in dogs that is primarily synthesized in the liver. It plays a role in the acute phase reaction of the non-specific immune system in mammals [1,2]. Normally, CRP is found in low serum concentrations in dogs. However, it rapidly increases in concentration as soon as 4 hours after the onset of an inflammatory response or tissue damage [3–6]. Elevated CRP has been observed in canine patients with surgical trauma, infectious diseases [7–9], neoplasia [10–12], and various other inflammatory diseases [6,13–17]. Therefore, canine CRP (cCRP) in serum is a useful and non-specific diagnostic marker for inflammatory conditions [15].

There are various methods available for detecting cCRP concentration in veterinary medicine [18–22], including enzyme-linked immunosorbent assay (ELISA) [23–25], latex agglutination tests [6,26], and immunoturbidimetric assay [27]. Among these, ELISA specific to cCRP has been validated [18] and is commercially available. However, this technique can be time-consuming and requires well-equipped laboratories. This makes them unsuitable for routine clinical use at the point-of-care test (POCT). To address these issues, rapid diagnostic tests have been developed. There are now commercial cCRP POCT available, including quantitative immunoassays that utilize an analyser [28,29], as well as semi-qualitative immunochromatography assays [30]. However, there are still drawbacks, such as inaccurate results. Therefore, further study is necessary to develop rapid and accurate kits for measuring cCRP concentration in serum.

The aim of this study was to develop a rapid and precise diagnostic test for the detection of canine serum CRP concentration, using a fluorescence immunoassay (FIA). We validated our diagnostic assay by testing clinical samples and comparing the results with those of a reference ELISA. Consequently, our goal was to offer a reliable and effective alternative to existing CRP detection methods in veterinary medicine.

## 2. Materials & methods

### 2.1. Development of FIA cCRP to measure cCRP in dogs

#### 2.1.1. Europium conjugation

The conjugates of europium nanoparticles and anti-cCRP mAb were prepared as previously described [31]. Initially, 1 mg of 200 nm europium nanoparticles were dissolved and activated in 1 mL of 4-morpholineethanesulfonic acid (MES) buffer (pH 6.1) containing 1.3 mM 1-ethyl-3(3-dimethylaminopropyl) carbodiimide hydrochloride and 100 mM *N*-hydroxysuccinimide under constant shaking (180 rpm) for 1 h at 25°C. After activation, the mixture was washed with MES buffer (pH 6.1) and centrifuged at 22,660 ×g for 15 min at 4°C. The supernatant was then aspirated, washed twice, and resuspended in MES buffer (pH 6.1) by sonication. Subsequently, 0.155 mg of specific anti-cCRP mAb was added, and the labelling reaction was performed for 2 h at 30°C with gentle, constant shaking at 100 rpm. After 2 h, the mixture was incubated with 20 μL of blocking buffer added and shaken at 30°C for 30 min. The uncoupled antibody was removed by centrifugation at 22,660 ×*g* for 15 min at 4°C. The washing process was repeated twice, and the conjugate was resuspended in 1 mL of storage buffer and stored at 4°C until use.

#### 2.1.2. Strip preparation for flow immunoassay

The mAb against cCRP was dispensed and immobilized at the appropriate positions (test line) on a nitrocellulose membrane (0.2–0.5 mg/mL). Goat anti-mouse IgG (1 mg/mL) (Arista Biologicals Inc., Allentown, PA, USA) was dispensed and immobilized on the control line of the membrane.

#### 2.1.3. Preparation for conjugate tube

The conjugate solution was mixed with 8% (w/v) europium-conjugated anti-cCRP, 2% (w/v) europium-conjugated rabbit IgG, 0.5 M NaCl, 1% (w/v) BSA, 5% (w/v) sucrose, and 0.1% (w/v) Tween20 in phosphate buffered saline at pH 7.4. The resulting solution was then dispensed into a tube and vacuum-dried for 1 h.

## 3. Strip reader

The Confiscope F20 reader is equipped with a 365 nm high-brightness Light Emitting Diode. A high-resolution camera is utilized to measure the generated fluorescent light. An optical filter, which can transmit only 615 nm of light, is also used. The reader consists of a power unit that supplies the required power, a control unit that calculates and quantifies fluorescence intensity, an input/output unit that displays results and receives operations, and an interface unit that can be connected to external devices such as Wi-Fi, Bluetooth, and USB.

## 4. Method validation

All assays used a recombinant cCRP (Cat. 8CC5, Hytest, Finland) as standard.

### 4.1. Linearity and precision

To assess accuracy of FIA cCRP, the linearity study performed with dilution of a standard cCRP antigen to concentrations of 200, 50, 15, 5, and 0 μg/mL using 1 × PBS. The assay was repeated three times for each concentration, and a standard curve was created by plotting the concentration of standard cCRP on the Y axis against the measured cCRP concentration by FIA cCRP on the X axis.

Precision was evaluated at three different levels of cCRP concentration: 5 μg/mL, 50 μg/mL, and 100 μg/mL. Intra- and inter-assay coefficients of variation (CVs) for the FIA cCRP were calculated using three batches from replicate measurements at each concentration. For the intra-assay CV, 10 replicate measurements were performed; for the inter-assay CV, we measured once a day for five days consecutively.

### 4.2. Limit of detection and limit of quantitation

We performed 20 measurements for each of the low concentration intervals (5.0 μg/mL, 4.0 μg/mL, and 2.0 μg/mL) and calculated the limit of detection (LOD) and the limit of quantitation (LOQ).

## 5. Ethics statement

This study was performed with approval (NON2023–108) from the Animal Care Committee of Jeonbuk National University. All dog owners signed informed consent. The blood samples were not exclusively collected for this study, but rather for diagnostic or health assessment purposes.

## 6. Clinical samples

A total of 194 canine serum samples were collected in Gyeonggi-do province, Korea from 2021 to 2022. The obtained serum from whole clotted blood through centrifugation was stored at −20°C for up to a week to measure cCRP concentration using FIA cCRP or at −80°C for up to three months for further analysis.

## 7. CRP analysis

**Figure 1.**
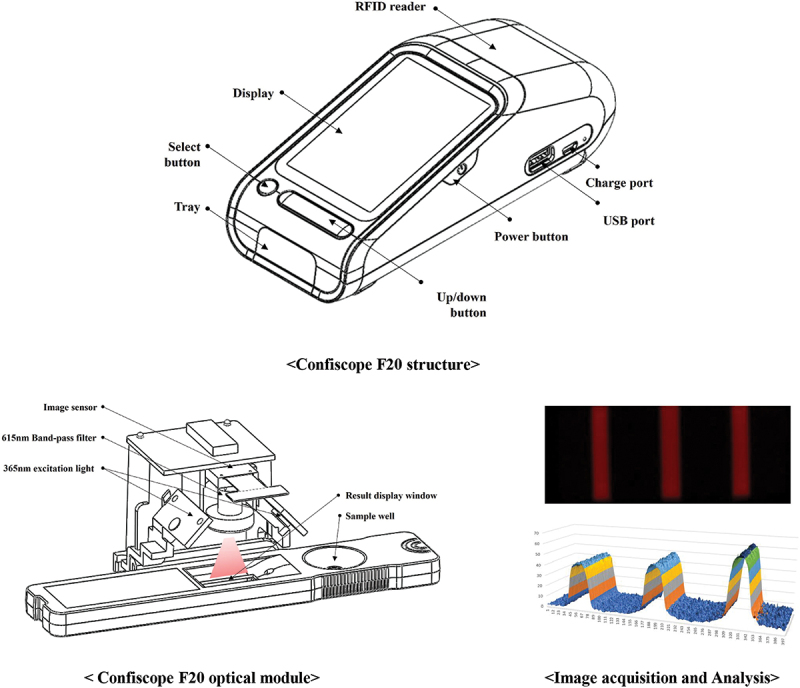
Schematic representation of strip reader. The Confiscope F20 is equipped with a high-brightness LED that emits 365 nm light. A high-resolution camera measures the generated fluorescent light using an optical filter that only transmits 615 nm of light. When the inspection begins, the internal optical module captures an image of the analysis area. The fluorescent signals generated in the reaction area are converted into digital signals through various image processing and mathematical calculation steps, and the result is displayed on the screen. LED, light emitting diode.

**Figure 2.**
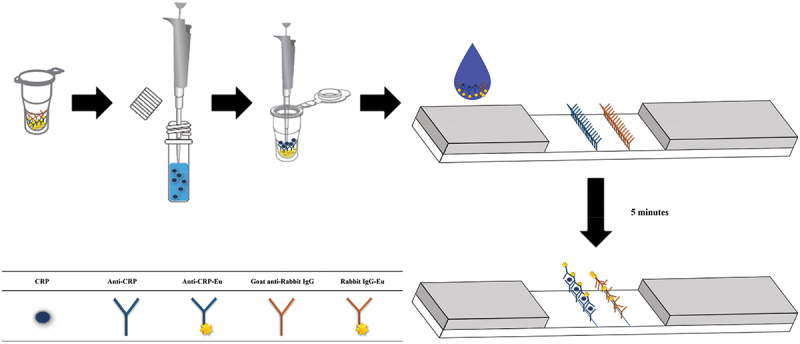
Schematic representation of FIA cCRP. FIA cCRP was designed as a medical device for *in vitro* diagnostic purposes that utilizes immunochromatography to quantitatively detect cCRP in dog serum or plasma, aiding in the diagnosis of inflammation. The device works by binding cCRP-specific antibodies to fluorescent particles such as europium in the fluorescent conjugate, as well as cCRP-specific antibodies bound to the nitrocellulose membrane test line. The fluorescent conjugate reacts primarily with cCRP in the sample to form an antigen-antibody complex, which moves along the membrane by capillary reaction. The complex then reacts with secondary cCRP antibodies to form an antigen-antibody complex that becomes immobilized on the line zone, providing a fluorescent signal strength that corresponds to the cCRP concentration present in the sample. The device is used with a dedicated analyser to measure cCRP levels. FIA, fluorescent immunoassay; cCRP, canine c-reactive protein.

All serum samples were analysed for FIA cCRP using a Confiscope F20 reader (Figures 1 and 2). To perform the test, 5 μL of serum was mixed with an assay diluent solution and thoroughly shaken. Next, 200 μL of the mixed sample was added to the conjugate tube and resuspended thoroughly. Then, 100 μL of the mixed sample was added to the sample hole of the test device and incubated. Finally, the results were analysed after 5 min.

To validate the diagnostic test, the cCRP concentrations measured through FIA cCRP were compared to those obtained using a commercially available ELISA kit (Cat. 41-CRPCA-E01, cCRP ELISA, ALPCO, Salem, NH, USA).

## 8. Statistical analysis

Statistical analysis was conducted using GraphPad Prism 5. The concentrations of cCRP measured by FIA cCRP and ELISA were statistically analysed and expressed as a mean, standard deviation (SD), and 95% confidence intervals (CI). To determine the correlation between the cCRP concentrations obtained by FIA cCRP and ELISA, linearity test and the Pearson correlation coefficient (Pearson’s ρ) was used. A Bland-Altman analysis was conducted to assess the agreement of the cCRP concentrations obtained by FIA cCRP and ELISA. The difference between the two measurements was calculated and plotted against the mean value of those measurements. A p-value less than 0.05 is considered to be significant.

## 9. Results

### 9.1. Limit of detection and limit of quantitation

We performed 20 measurements for each of the low concentration intervals (5.0 μg/mL, 4.0 μg/mL, and 2.0 μg/mL) and calculated the limit of detection (LOD) and the limit of quantitation (LOQ). Our results showed that FIA cCRP had an LOD of 4.0 μg/mL and a LOQ of 5.0 μg/mL.

### 9.2. Accuracy and precision

**Figure 3.**
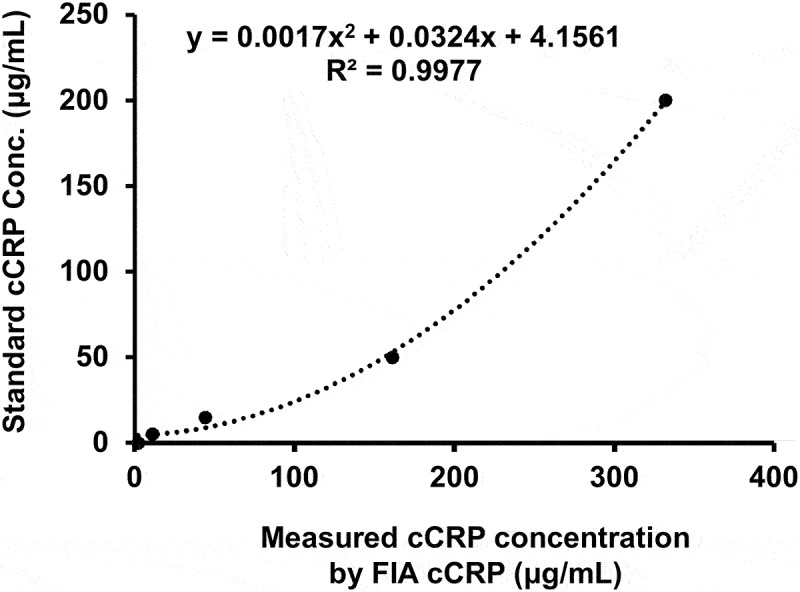
Linearity curve of measured value for cCRP using FIA cCRP. R^2^ was 0.9977. cCRP, canine c-reactive protein; Conc., concentration; FIA, fluorescent immunoassay.

To assess the accuracy of FIA cCRP, the linearity study performed with dilution of a standard cCRP antigen to concentrations of 200, 50, 15, 5, and 0 μg/mL using 1 × PBS. The assay was repeated three times for each concentration, and a standard curve was created by plotting the concentration of standard cCRP on the Y axis against the measured cCRP concentration by FIA cCRP on the X axis. In Figure 3, the linearity test showed a strong correlation with an R^2^ value of 0.9977 (concentration range of 0–200 μg/mL). The precision study was carried out for standard cCRP concentrations ranging from 5 μg/mL to 200 μg/mL using three batches. The results obtained an intra-assay CV < 13% and an inter-assay ≤13.8%, respectively, from replicate CRP measurment (Table 1).

**Table 1.** For three different concentrations of standard cCRP, the intra-assay CV was measured by 10 replicates, and the inter-assay CV was measured once a day for five consecutive days. The intra- and inter-assay CVs measured by FIA cCRP meet the quality specifications, as the values are below the _min_CV of 18.2% [35]. cCRP, canine c-reactive protein; CV, coefficient of variation; FIA, fluorescent immunoassay; _min_CV, minimum CV; SD, standard deviation.

Standard cCRP concentration	Intra-assay CV			Inter-assay CV		
	Mean (μg/mL)	SD (μg/mL)	CV (%)	Mean (μg/mL)	SD (μg/mL)	CV (%)
5 μg/mL	4.78	0.62	12.96	4.82	0.67	13.80
50 μg/mL	50.91	3.10	6.09	49.23	2.47	5.02
200 μg/mL	200.28	5.49	2.74	199.01	4.70	2.36

### 9.3. cCRP analysis

To assess the FIA cCRP, we analysed all 194 canine serum samples. The results showed that serum cCRP values ranged from 2.58 to 309.78 μg/mL (median 40.52 ± SD 73.66 μg/mL). Table 2 shows the mean, SD, and 95% CI for different cCRP concentration intervals: cCRP level 1 (<20 µg/mL, *n* = 7), cCRP level 2 (20–100 µg/mL, *n* = 6), and cCRP level 3 (>100 µg/mL, *n* = 8). To validate these results, we selected 21 samples out of the total 194 and performed ELISA. The ELISA results showed that cCRP values in serum ranged from 8.02 to 232.05 μg/mL (median 62.80 ± SD 77.59 μg/mL). In 18 out of 21 specimens, the FIA cCRP results matched the ELISA cCRP concentrations both above and below 20 μg/mL. However, in three samples where the FIA cCRP value was less than 20 μg/mL (6.19 μg/mL, 16.33 μg/mL, and 17.99 μg/mL), the ELISA measured cCRP values were greater than 20 μg/mL (25.48 μg/mL, 54.73 μg/mL, and 27.81 μg/mL).

**Table 2.** A total of 21 canine samples were measured using FIA cCRP with three different concentration intervals: <20 μg/mL (low), 20–100 μg/mL (moderate), and >100 μg/mL (high). Data are mean, SD, and 95% CI for each serum cCRP level. FIA, fluorescent immunoassay; cCRP, canine c-reactive protein; SD, standard deviation; CI, confidence interval.

	Serum cCRP Level 1 (< 20 μg/mL, *n* = 7)	Serum cCRP Level 2 (20–100 μg/mL, *n* = 6)	Serum cCRP Level 3 (> 100 μg/mL, *n* = 8)
Mean	11.38	61.72	151.96
SD	4.38	22.3	16.2
95% CI	7, 15.8	39.4, 84	136, 168

### 9.4. Comparison of FIA cCRP and ELISA

**Figure 4.**
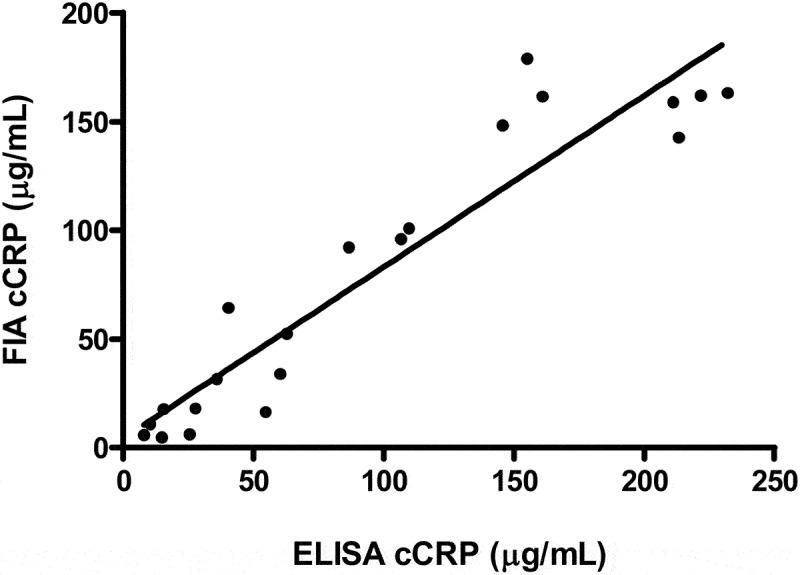
Correlation between the FIA cCRP and ELISA. Correlation coefficient (Pearson’s ρ) was 0.94 (*p* < 0.001; *n* = 21). FIA, fluorescent immunoassay; cCRP, canine c-reactive protein; ELISA, enzyme-linked immunosorbent assay.

**Figure 5.**
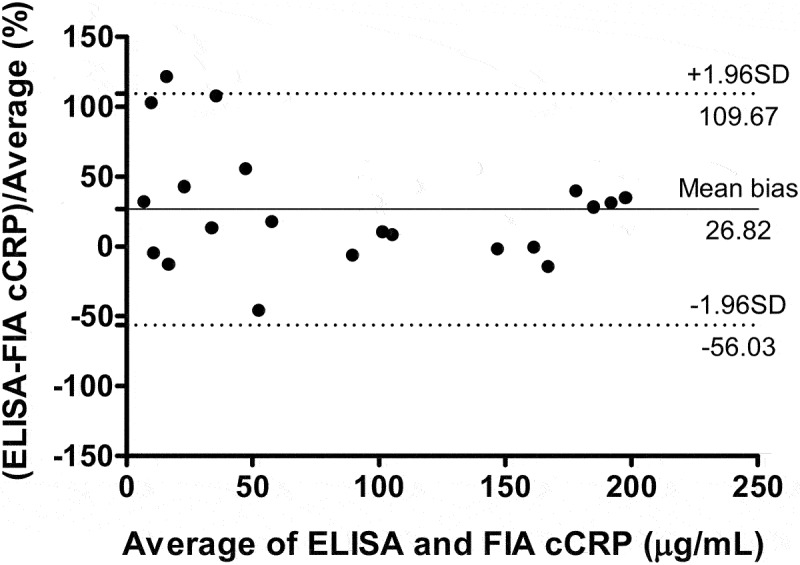
Bland–Altman difference plot. Bias was 15.9 (SD ±27.4, 95% limits of agreement between − 37.77 and 69.58). SD, standard deviation; FIA, fluorescent immunoassay; cCRP, canine c-reactive protein; ELISA, enzyme-linked immunosorbent assay.

To analyse the relationship between FIA cCRP and ELISA cCRP concentrations in serum, we calculated Pearson's correlation coefficient and created Bland–Altman plot. The correlation coefficient (Pearson’s ρ) between FIA cCRP and ELISA was 0.942 with a 95% CI of 0.859 to 0.976 for the 21 specimens (Figure 4). The calculated correlation was statistically significant (*p* < 0.001). In the Bland-Altman difference plot, bias was 26.8% ± 42.3%, and 95% limits of agreement was from −56.0% to 109.7% (Figure 5).

## 10. Discussion

Canine CRP is a useful marker for diagnosing and monitoring various diseases in veterinary medicine [32]. While elevated levels of cCRP are non-specific for disease, they are more effective and sensitive for detecting inflammatory responses than traditional inflammation markers, such as leukocytosis and left shifts in neutrophils [15]. However, previous methods for detecting cCRP levels in dog serum have been limited the ability to provide rapid clinical analysis. ELISA, one of the most commonly used methods, requires a well-equipped laboratory and is time-consuming, as well as requiring analysis of samples in batches. In this study, we evaluated a new rapid and quantitative POCT based on FIA for measuring cCRP serum concentration and compared this assay to a previously validated ELISA.

The FIA cCRP demonstrated excellent linearity, with an R-squared value of 0.9977 across a range of 0–200 μg/mL. These findings are consistent with a previous study that evaluated other quantitative diagnostic tests for cCRP [33,34]. In the precision study, both inter- and intra-assay CVs were found to be less than 14%, which is lower than the minimum CV of 18.2% [35], indicating that the FIA cCRP met the quality criteria for CV. The LOQ established in this study was 5.0 μg/mL, which is similar to the immunoturbidimetric assay (4 mg/L) but lower than other POCTs (10 mg/L) for cCRP [34,36]. These results suggest that the FIA cCRP offers strong linearity and precision and may provide increased sensitivity compared to previously available POCTs.

In general, a correlation coefficient of >0.9 is considered to indicate a very high positive correlation between two variables [37]. In this study, Pearson’s ρ was found to be 0.94, indicating a strong positive correlation between the results of the FIA cCRP and ELISA. The Bland-Altman plot is a useful tool for calculating the agreement between two methods [38]. In this study, the plot revealed a constant bias in the results from FIA cCRP. These results tended to be lower than those of the ELISA. Although a small discrepancy was observed at low CRP concentrations (<20 μg/mL), most of the data fell within the 95% limits of agreement.

One limitation of our study is that all samples were stored at −80°C for up to three months. However, a previous study has reported that cCRP remains stable at −10°C for at least four months [39], and another study revealed that cCRP is relatively stable at −70°C for four years [20]. Additionally, there is no effect on results up to four freeze-thaw cycles [40]. Hence, it is unlikely that our results were influenced by freezing during storage.

In conclusion, our findings indicate that the new FIA cCRP method is fast and reliable for measuring serum cCRP concentration in dogs. Therefore, we suggest that it could be suitable for clinical use, such as early diagnosis and therapeutic monitoring.
